# CALGB 140503 and the shift to sublobar resection for small, peripheral, node-negative NSCLC: historical context, secondary analyses, and next steps

**DOI:** 10.3389/fonc.2026.1791539

**Published:** 2026-04-10

**Authors:** Oliver S. Chow, David R. Jones, Thomas E. Stinchcombe, Nasser K. Altorki

**Affiliations:** 1 Division of Thoracic Surgery, Department of Cardiothoracic Surgery, Weill Cornell Medicine, New York-Presbyterian Hospital, New York, NY, United States; 2 Division of Thoracic Surgery, Department of Surgery, Memorial Sloan-Kettering Cancer Center, New York, NY, United States; 3 Duke Cancer Institute, Duke University Medical Center, Durham, NC, United States

**Keywords:** CALGB 140503, early-stage lung cancer, lobectomy, lung cancer, sublobar lung resection

## Abstract

The surgical management of early-stage non–small cell lung cancer (NSCLC) has been reshaped by contemporary randomized data supporting lung-sparing approaches in carefully selected patients. CALGB/Alliance 140503, a multicenter phase III trial, compared sublobar resection (wedge or segmentectomy) with lobectomy for peripheral, clinically node-negative NSCLC ≤2 cm, randomizing patients only after rigorous intraoperative nodal assessment. Sublobar resection proved noninferior to lobectomy with respect to disease-free survival, with comparable overall survival and recurrence patterns. Alongside other important randomized trials like JCOG 0802/WJOG 4607L, sublobar resection has now been established as an acceptable – and for some patients preferable – strategy for stage IA (≤ 2 cm) NSCLC. Beyond its primary results, CALGB 140503 has generated a series of secondary and exploratory analyses that continue to refine day-to-day clinical decision-making. This mini-review aims to synthesize the current state of insights from this trial, highlighting ongoing controversies and key gaps for future investigation that will further optimize the management of early-stage NSCLC.

## Introduction

Lobectomy has been the surgical standard of care for the treatment of early-stage lung cancer since the early 1960s (1). Due to a rising interest in segmental resection in the 1970s (2), the Lung Cancer Study Group (LCSG), a newly minted National Cancer Institute cooperative group, conducted a seminal randomized trial comparing lobectomy with limited resection in patients with clinical T1N0 NSCLC (3).

Patients were eligible for the trial if they had clinical T1N0 peripheral tumors (3 cm or less) and no evidence of systemic disease based on their history, clinical examination, and normal blood chemistries. Preoperative computed tomographic (CT) examinations were not mandated nor routine in that era. Patients were intraoperatively randomized to either lobectomy (125 patients) or limited resection (122 patients). Both wedge resection and anatomical segmentectomy were allowed as modalities of limited resection, which is now more commonly referred to as sublobar resection (SLR). In 1995, the authors reported a 50% increase in deaths from lung cancer after SLR and concluded that lobar resection is favored in this setting. However, the observed differences in death rates were of borderline statistical significance. Importantly, locoregional recurrence exclusive of second primaries was observed in 8 patients in the lobectomy group and 21 patients in the SLR group. This tripling of locoregional recurrence after SLR further favored lobectomy as the treatment of choice. Despite its significant limitations, the LCSG trial established the surgical canon for early-stage lung cancer, and its results went unchallenged for nearly three decades.

Since the early 2000s, there has been a rising interest in revisiting the role of SLR in early-stage lung cancer (4). This was primarily driven by the significant advances in imaging modalities, improved clinical staging as well as the widespread adoption of CT into routine practice and for lung cancer screening with a resultant increase in the detection of early stage NSCLC. Two large, randomized trials initiated in the early 2000s tested the hypothesis that SLR is non-inferior to lobectomy in a subset of early-stage node-negative NSCLC (5, 6). The North American trial (CALGB/Alliance 140503) (5) randomly assigned patients to lobar or sublobar resection (including wedge resection), whereas the Japanese trial (JCOG 0802/WJOG 4607L) (6) randomized patients to lobectomy or anatomical segmentectomy. This review synthesizes the expanding body of published evidence derived from the CALGB 140503 trial and examines its implications for “real-world” practice and future investigation.

### CALGB/Alliance 140503: trial design and primary outcome

**Figure 1:**
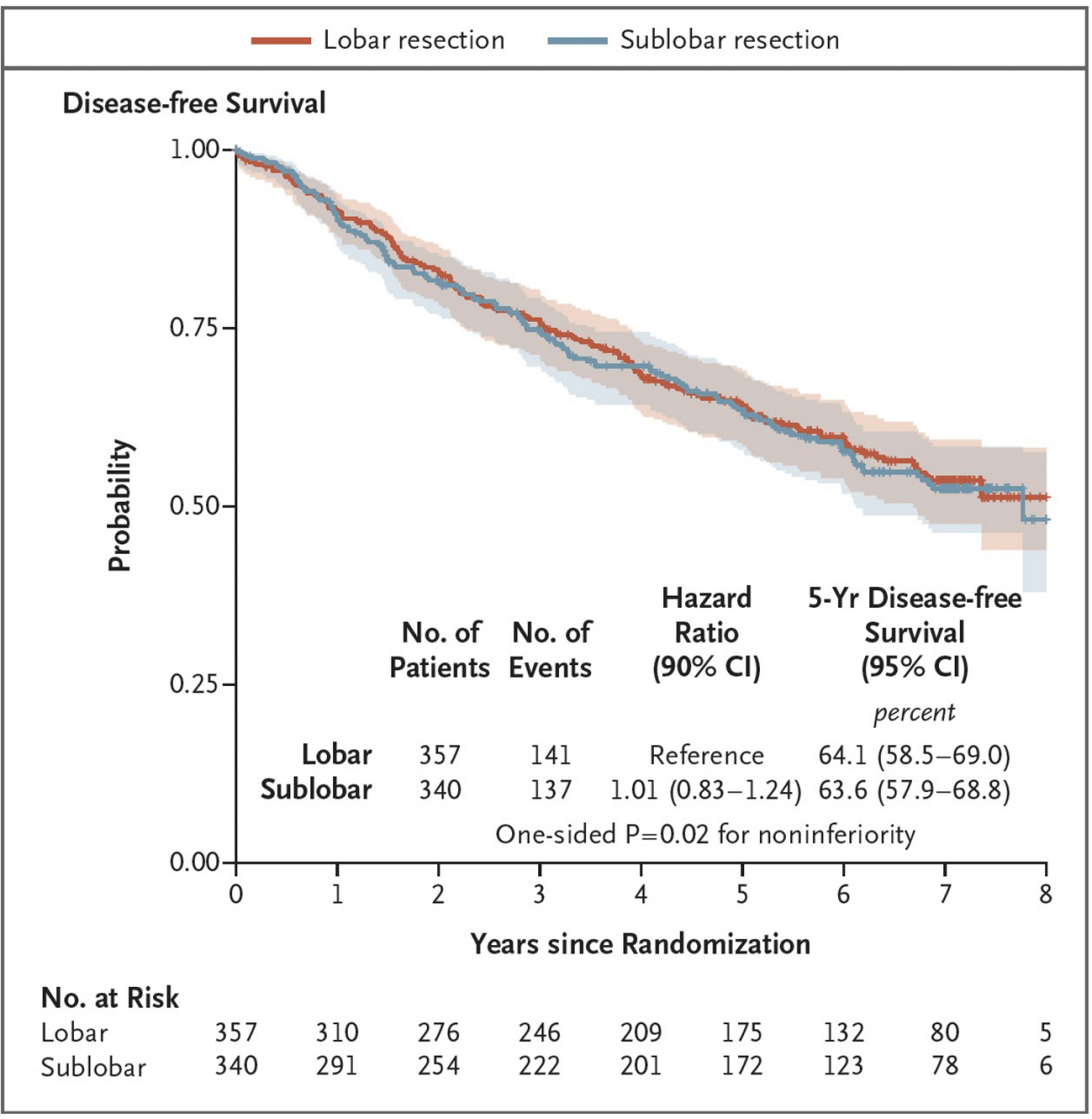
Primary end point in CALGB 140503: Disease-free-survival after lobar (red) or sublobar resection (blue) of clinically-staged T1aN0 ≤ 2 cm peripheral tumors after intraoperative or preoperative confirmation of node-negative disease. Reprinted from Altorki N, Wang X, Kozono D, et al. *N Engl J Med* 2023;388:489-98, Copyright © 2023 with permission from Massachussetts Medical Society.

In 2005, the Cancer and Leukemia Group B (subsequently the Alliance for Clinical Trials in Oncology) initiated a prospective, randomized, non-inferiority trial comparing lobar and SLR (wedge resection or segmentectomy) in patients with clinically node-negative peripheral lung cancers two centimeters or less in size. Randomization occurred intraoperatively only after frozen section confirmed the absence of metastatic disease in two mediastinal and one major hilar nodal stations. Nodes sampled within 6 weeks of surgical resection by mediastinoscopy or EBUS did not need to be resampled. The trial’s primary endpoint was disease-free survival, with several secondary endpoints including overall survival, recurrence patterns, and changes in pulmonary function following resection. The trial was activated in 2007 and completed its target accrual in 2017. Nearly 697 patients were randomized to either lobar or SLR by 125 surgeons at 83 institutions across the United States, Canada and Australia. Remarkably, 80% of all resections were performed minimally invasively, with a 30-day mortality of 1.1% after lobectomy and 0.6% after sublobar resection. Adverse events of grade 3 or worse occurred in 15% of patients assigned to lobar resection and 14% of patients assigned to sublobar resection. After a minimum follow-up of five years and a median follow-up of seven years, the trial met its primary endpoint of non-inferiority of disease-free survival following SLR compared to that after LR (hazard ratio for disease recurrence or death, 1.01). Five-year disease-free survival was 63.6% after SLR and 64.1% after LR (**Figure 1**). Five-year overall survival was 80.3% after SLR and 78.9% after LR. Disease recurrence developed in 30.4% of patients after SLR and 29.3% after lobectomy. Locoregional recurrence occurred in 13.4% after SLR and 10.0% after lobectomy. More than 50% of the recurrences in each group were systemic in nature. Although the reduction in FEV1 and FVC was numerically better after sublobar resection, the intergroup differences in both parameters were neither statistically significant nor clinically meaningful. The investigators concluded that sublobar resection is an acceptable alternative to lobectomy in this highly selected cohort of patients. Most surprising was the 30% incidence of overall recurrence in both groups, suggesting the clinical imperative for including these patients in neoadjuvant or adjuvant trials to potentially improve outcomes.

### Survival and recurrence patterns after wedge resection and segmentectomy

In the aftermath of the LCSG trial, the prevailing wisdom was that anatomical segmentectomy with individual ligation of segmental vasculature and bronchi is oncologically superior to wedge resection. This led to significant criticism of the CALGB 140503 trial design since both segmentectomy and wedge resection were allowed modalities of sublobar resection. However, the decision to allow wedge resections was made after a thorough review of large national registry data showed that, despite the results of the LCSG trial, wedge resections were the most commonly performed modality of SLR. Therefore, the study team favored allowing wedge resections so that the trial reflected “real-world” settings and to provide some quality-controlled evidence that would either refute or support its potential efficacy as an oncologic resection.

**Figure 2:**
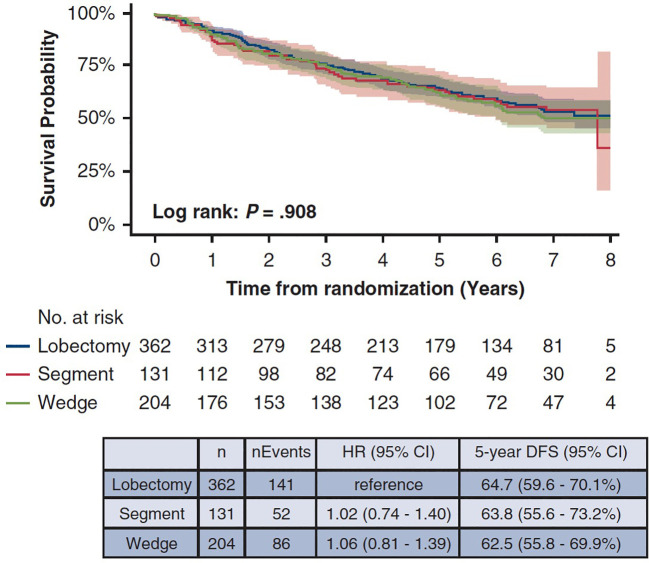
Disease-free-survival after lobectomy (blue), segmentectomy (red), and wedge resection (green) in CALGB 140503. Reprinted from Altorki NK, Wang X, Damman B, Mentlick J, Landreneau R, Wigle D, et al. *J Thorac Cardiovasc Surg* 2024;167:338–347 Copyright © 2024 with permission from Elsevier.

In a *post-hoc* analysis, the investigators reported the differences in oncologic outcomes based on the extent of SLR (204 wedge resections and 131 segmentectomies) (7). Five-year DFS was 63.8% after segmentectomy, and 62.5% after wedge resection (P = 0.888, log-rank test, see **Figure 2**). Five-year lung cancer-specific survival was 86.8% after lobar resection, 89.2% after segmental resection, and 89.7% after wedge resection. Although locoregional recurrence was numerically lower after segmentectomy (12% vs 14%; P = 0.295), freedom from locoregional recurrence at 5 years was 84.6% after segmentectomy and 82.7% after wedge resection. The investigators concluded that both wedge resection and anatomic segmentectomy are acceptable treatment options for patients with peripheral T1a NSCLC without metastasis to the hilar and mediastinal nodes. There are several important caveats to consider before accepting this conclusion. First, in contrast to usual clinical practice, the trial protocol suggested an “adequate” surgical margin of 2 cm around the tumor or a margin equivalent to the tumor size. Analysis of the surgical margins revealed a significant difference in the median margin length, favoring segmentectomy (2 cm vs. 1.6 cm, P = 0.031). Still, the median margin length for wedge resection was larger than the median tumor size (1.5 cm) in that group. Second, intraoperative frozen section examination of the margin of resection, another uncommon clinical practice, was performed in 281 of 335 (84%) patients undergoing sublobar resection. Ten patients had positive margins on frozen section after sublobar resection; 8 after wedge resection (5 underwent additional wedge resection, 3 underwent lobectomy), and 2 after segmentectomy (1 underwent extended segmentectomy, the other underwent lobectomy). None of these patients developed subsequent disease recurrence in the trial follow-up. Despite these reassuring results, the CALGB 140503 trial was not powered to test for noninferiority between the two SLR modalities. Furthermore, patients were not randomly assigned to the type of sublobar resection, raising the possibility of unrecognized patient selection bias.

In summary, the results of this *post-hoc* analysis challenged the long-held notion that wedge resections are inherently oncologically suboptimal. In the absence of a randomized trial directly comparing the two modalities, the results from CALGB 140503 are currently the best available evidence suggesting that oncologic outcome is not impacted by the type of sublobar resection for highly-selected patients with peripheral ≤ 2 cm tumors with no nodal disease.

### Patients with small peripheral node-negative lung cancer have a significant risk for second primary lung cancer

It is recognized that patients who undergo curative resection for NSCLC are at a lifetime risk for developing second primary lung cancers (SPLC). Current estimates of SPLC are based on retrospective case series or registry data that include a heterogeneous patient population, where the modality, frequency, and duration of surveillance imaging were not standardized. CALGB 140503 included a relatively homogeneous population with peripheral node-negative tumors ≤ 2 cm in size. Surveillance was initially done by CT scan every 6 months for the first year, followed by annual scans for 5 years, which was later amended to the contemporary standard of every 6 months for the first 2 years, followed by annual scans. The determination of a SPLC was made by the treating physician and recorded in the trial database. The clinical criteria for diagnosis of SPLC were a tumor with a different histology, a tumor diagnosed 2 years after the index cancer, or a tumor of a similar histology located in a different lobe or segment without intervening nodal metastases. These criteria preceded the use of next-generation sequencing.

In a secondary analysis performed two years after random assignment (8), the rate of SPLC per patient per year was 3.4% for the whole study population with a cumulative incidence of 15.9% (95% CI: 12.9-18.9) at five years. The rate per patient per year in the SLR and the LR arm was 3.8% (95% CI: 2.9-4.9) and 3.1% (95% CI: 2.4-4.1), respectively. The estimated 5-year cumulative incidence of SPLC in the study population, SLR arm, and LR arm was 15.9% (95% CI: 12.9-18.9), 17.2% (95% CI: 12.7-21.5), and 14.7% (95% CI: 10.6-18.7), respectively. While sublobar resection patients had a slightly higher rate of SPLC than lobar resection patients (17.2% vs. 14.7%), this was not a statistically significant difference and likely reflects a larger remaining extent of parenchyma at risk. The rate of SPLC observed in this select cohort of patients is clinically significant and is almost 50% higher than previously reported. The risk appears unabated as late as seven years following resection, raising questions about the optimal frequency, duration, and modality of surveillance.

### How much lymph node dissection is “enough”?

The optimal extent of lymphadenectomy for clinical stage IA NSCLC remains controversial. This debate has persisted even though at least two randomized trials have not demonstrated a survival benefit after complete lymph node dissection compared with systematic lymph node sampling in clinical T1N0 tumors (9–11). The ACOSOG Z0030 trial, published in 2011, looked specifically at whether complete mediastinal lymph node dissection improved oncologic outcomes compared to systematic sampling in patients who had T1-2, N0 or non-hilar N1 NSCLC (9). In that trial, patients with eligible tumors underwent systematic nodal sampling of three N2 and one N1 stations. If there was no evidence of nodal metastasis on frozen section examination, patients were randomized to either no further lymph node removal or complete lymph node dissection. In those who received complete lymph node dissection after negative systematic sampling, only 4% (21/525) were found to have occult N2 disease. Disease-free and overall survival did not differ between these groups, suggesting that as long as systematic sampling has been accomplished in patients with early-stage NSCLC, additional lymph node dissection does not further improve oncologic efficacy.

In CALGB 140503, the extent of lymph node dissection was left to each surgeon’s discretion, but eligibility required confirmation of N0 status by frozen section examination from at least two N2 stations and one major hilar (N1) nodal station. Due to the inherent variability in surgical practice patterns, the trial investigators conducted an unplanned secondary analysis to examine whether the extent of nodal dissection or sampling was associated with differences in survival (12). Most patients (51%) underwent systematic nodal sampling (more than 2 N2 stations and 1 N1 station), 26% had complete lymph node dissection (CLND, near-complete removal of all nodal tissue in upper, middle, and lower mediastinal stations), and 23% underwent simple sampling alone, which was the minimum requirement for eligibility. There was no difference in disease-free or overall survival between lobar and sublobar resection based on the extent of node dissection. There was also no difference in survival within each arm of the trial based on the extent of nodal dissection. The rates of occult nodal disease were low (3.3% occult N1 and 1.3% occult N2), and differences in survival based on the extent of nodal dissection were not statistically significant. Additionally, most recurrences were systemic, regardless of the extent of lymph node assessment performed. Overall, these findings suggest that for patients who are carefully selected and staged preoperatively and/or intraoperatively, more extensive nodal clearance does not offer an oncologic benefit.

These conclusions need to be interpreted in the context of well-recognized inconsistency in lymph node evaluation in real-world practice. In a large population database study (13), Smeltzer, et al. stratified 2047 patients who underwent curative intent surgical resections into groups of increasing stringency of lymph node dissection; from lung resections with no nodes assessed to those with R0 resections and examinations of at least three mediastinal lymph node stations, at least one N1 lymph node station, and at least ten lymph nodes altogether (reflecting a combination of the NCCN and American College of Surgeons Commission on Cancer recommendations). They found that increasing stringency of lymph node dissection was associated with greater differentiation in overall survival between pN0, pN1, and pN2 disease. But in their large cohort, only 26.6**%** of patients met the most rigorous nodal assessment criteria.

Taken together, the trials and studies addressing lymph node evaluation consistently underscore a key principle: meticulous and accurate nodal assessment remains integral to providing optimal oncologic outcomes. Once that standard has been met, a more extensive lymphadenectomy does not appear to provide additional benefit for patients with peripheral clinically node-negative NSCLC.

### Visceral pleural invasion is associated with high recurrence rates and worse survival outcomes that are not mitigated by lobar resection

Although visceral pleural invasion (VPI) has long been associated with recurrence and worse survival in NSCLC, its negative prognostic impact in patients with small tumors (≤ 2 cm) has not been definitively shown. Given the uncertainty about the prognostic relevance of VPI in these small tumors, some thoracic surgeons have advocated for lobar resection if visceral pleural invasion is suspected intraoperatively. In a *post-hoc* exploratory secondary analysis, 113 out of 697 (16%) randomized patients in CALGB 140503 had tumors with VPI (pT2) (14). Five-year disease-free survival was 53.3% (95% CI: 61.9%-70.2%) in patients with pT2 compared with 65.9% (95% CI: 44.3%-64.1%) in patients with pT1 tumors (stratified log-rank: P = 0.02). Disease recurrence developed in 41.6% of patients with pT2 and 27.6% of those with pT1. Five-year recurrence-free survival was 58.2% (95% CI: 69.2%-77.1%) for pT2 tumors and 73.1% (95% CI: 49.2%-68.8%) for pT1 tumors (stratified log-rank: P = 0.01). Importantly, there were no intergroup differences in disease-free or recurrence-free survival based on the extent of parenchymal resection. The investigators concluded that although patients with VPI had worse survival and a higher rate of recurrence compared to patients with pT1 tumors, oncological outcomes were independent of the extent of parenchymal resection. Once again, these data support the inclusion of these patients in adjuvant therapy trials.

### Ongoing controversies and knowledge gaps

#### Wedge vs. segmentectomy for solid nodules

Many thoracic surgeons consider segmentectomy an oncologically superior procedure compared to wedge resection. While segmentectomy is commonly associated with longer resection margins and better assessment of N1 nodal stations, there is no definitive evidence that it is associated with better survival than wedge resection in properly selected patients. To date, the only existing trial evidence was based on the results of the Lung Cancer Study Group trial, which compared lobectomy with limited resection. Among the 124 patients in the limited resection arm, 82 underwent a segmentectomy and 40 underwent a wedge resection. Although there was a tripling of local recurrence in the limited resection arm, the authors stated “this effect appears to apply regardless of whether the histology was squamous or non-squamous or whether the intended resection was wedge or segmental resection” (3). Despite the apparent similarity in oncological outcomes between the two procedures reported by CALGB 140503 investigators, the numerically higher event rate after wedge resections and the results from many retrospective case series have kept the debate alive. In the absence of adequately powered randomized trials directly comparing the two modalities of SLR, thoracic surgeons will determine the optimal sublobar resection strategy on a case-by-case basis, balancing surgical margin, nodal assessment, pulmonary reserve, and oncologic efficacy.

#### Lobar or sublobar resection in patients with high-risk histological characteristics

Another knowledge gap area is the impact of high-risk pathological characteristics on survival and recurrence, and whether that effect is modifiable by a more extensive parenchymal resection. Some of these characteristics, such as cell type (squamous vs. non-squamous), VPI, and lymphovascular invasion (LVI) were recognized at the time of trial design, but not considered prognostically relevant. Other features recognized since then include spread through the air spaces (STAS) and specific histological patterns of adenocarcinoma, such as the micropapillary and solid subtypes. The impact of these variables – individually or collectively – on prognosis and their potential interaction with the extent of resection is a subject of intense interest and ongoing studies. As discussed previously, CALGB 140503 investigators reported that VPI is a powerful prognostic indicator that is associated with worse DFS and RFS regardless of the extent of lung resection. A more comprehensive study was presented by Yatabe and colleagues (IASLC, Barcelona 2025), who performed an exhaustive retrospective central histopathological review of 642 specimens resected in JCOG 0802 (lobectomy:322; segmentectomy: 318). Their results, yet unpublished, showed that STAS was observed in 726 patients (49%), LVI was observed in 319 patients (21%), and VPI was observed in 138 patients (9%). The incidences of STAS and LVI were higher in the micropapillary-predominant tumors, whereas VPI was mainly observed in the solid-predominant tumors. Interestingly, the incidence of local recurrence in patients with STAS was 16.7% after lobectomy and 17.6% after segmentectomy compared to 6% in each arm in the absence of STAS. The presence of STAS was identified as an independent prognostic factor for both RFS and OS, regardless of the extent of resection. Collectively, these results highlight the prognostic relevance of STAS, LVI, and VPI, as well as micropapillary and solid histologic subtypes in the assessment of risk of recurrence in patients with resected stage I lung adenocarcinoma. A similar analysis of resection samples from patients enrolled in CALGB 140503 is in the early planning phase.

#### Does sublobar resection preserve pulmonary function?

To date, three randomized trials (LCSG 821 (3), CALGB 140503 (5), JCOG 0802 (6)) have all shown that compared to lobar resection, the percentage reduction in forced expiratory flow rates (FEV1 and FVC) following sublobar resection was neither statistically significant nor clinically meaningful. Unfortunately, these are static measurements that may not directly correlate with functional capacity. A more detailed analysis of the preservation of pulmonary reserve might be better assessed by more functional evaluation, such as the 6-minute walk test or cardiopulmonary exercise testing, and perhaps most importantly, a detailed longitudinal analysis of patient-reported outcomes.

### CALGB 140503 in context with JCOG 0802 and DRKS00004897

Three contemporaneous randomized trials (CALGB 140503 (5), JCOG 0802 (6), and DRKS00004897 (15)) comparing sublobar resection with lobectomy have collectively redefined the surgical standard for peripheral ≤2 cm NSCLC. Although this review does not aim to directly compare these trials – and cross-trial comparisons should be interpreted with caution – it is worth highlighting several key differences and shared conclusions. CALGB 140503 permitted both anatomic segmentectomy and wedge resection as sublobar resection techniques, reflecting real-world practice in North America and Europe, where wedge resection remains the most common form of sublobar resection. The cohort included a larger proportion of smokers (> 90%) and more squamous cell carcinomas. The inclusion criteria was also determined based on the size of the solid component of the tumor. In contrast, JCOG 0802 only allowed anatomic segmentectomies, consisted of a predominantly East Asian cohort with nearly 50% never-smokers and close to 90% adenocarcinomas. JCOG 0802 enrolled tumors ≤ 2cm and initially required a consolidation-to-tumor ratio of ≥ 0.25, but amended this threshold to ≥ 0.5 following the results of JCOG 0201, which demonstrated very low relapse rates among predominantly non-solid tumors. Still, this resulted in nearly 50% of the tumors in the final cohort being part-solid. Despite these differences in operative technique, patient demographics, and tumor biology, the oncologic conclusions converged. CALGB 140503 demonstrated non-inferior disease-free survival and overall survival for sublobar resection versus lobectomy. JCOG 0802 showed a statistically significant, albeit modest, overall survival advantage with segmentectomy compared with lobectomy (HR 0.66, 95% CI 0.47-0.93), but no significant difference in disease-free survival. The overall survival benefit after segmentectomy appeared to be driven by a nearly two-fold higher rate of non-lung cancer-related deaths in the lobectomy group. This has led to speculation that lung parenchymal preservation offers retention of long-term cardiopulmonary fitness; yet this remains unproven, especially given the relatively modest differences in the measured change of pulmonary function between groups within the trials. The German DRKS00004897 study, though smaller (n=107), demonstrated non-inferior overall survival after segmentectomy compared with lobectomy, but also added important quality-of-life assessments as a primary outcome using the EORTC QLQ-C30 and the QLQ-LC13 surveys at 6 weeks, 3 months, 6 months, and 12 months after surgery (16). They reported improved physical and social function recovery after segmentectomy compared with lobectomy, with most measures returning to baseline between 3 and 6 months. By contrast, dyspnea, fatigue, and cognitive function had not returned to baseline 12 months after lobectomy.

Across distinct patient populations, histologic distributions, and surgical approaches, a unifying message has emerged: In properly selected patients with small, peripheral, node-negative tumors, more extensive surgical resection does not confer oncologic benefit, and might come at the cost of physiologic reserve and quality of life. Furthermore, with ongoing advances in radiographic surveillance and expanding options to diagnose and treat new primary or recurrent disease, the goal of lung preservation should not be casually dismissed, and more scrutiny should be held to lobectomy as the former default option for these tumors.

### The elephant in the room

Perhaps the most glaring finding of CALGB 140503 is the unexpectedly high incidence of recurrence observed in both arms of the trial. Interestingly, more than 50% of these recurrences were systemic in nature, which provides compelling evidence to include these patients in neoadjuvant or adjuvant trials to improve survival. The challenge is identifying those patients most likely to recur, such that the majority of patients can be spared unnecessary and potentially harmful interventions. Unfortunately, molecular biomarkers such as circulating tumor DNA are associated with low sensitivity in these small tumors. Other potential biomarkers include radiomics or peripheral immune-based biomarkers, both of which are in their infancy but may hold future promise. The only currently available off-the-shelf biomarkers are the high-risk pathological characteristics previously discussed and which occur or co-occur in 30-40% of patients. A recently activated trial in this space was TROPION-LUNG12, a global phase III adjuvant trial in patients with completely resected stage I adenocarcinoma without actionable genomic mutations (17). The trial aimed to randomize 660 patients who are either ctDNA-positive or have high-risk pathological features 2:1:2 to adjuvant detumomab deruxtecan followed by rilvegostomig (a bispecific antibody binding PD-1 and TIGIT), adjuvant rilvegostomig monotherapy alone, or standard of care. Unfortunately, the trial was terminated early due to poor accrual, likely due to stringent inclusion criteria and a potentially challenging two-step design. In the recently reported AIM-HIGH trial (18), investigators risk-stratified patients with completely resected stages IA-IIA non-squamous cancers using a validated PCR-based 14-gene assay. Patients with a molecular high risk were randomly assigned (1:1) to four cycles of platinum-based adjuvant chemotherapy or observation. At a pre-specified interim analysis, 24-month disease-free survival was 96% with adjuvant chemotherapy versus 79% with observation (hazard ratio 0·22 [0·06–0·76]; P = 0·0087). The principal finding of this trial was the validation of the 14-gene assay in identifying patients with molecular high risk who benefited from adjuvant chemotherapy. Though promising, these results need to be validated in a larger independent cohort including patients treated with adjuvant chemoimmunotherapy. These two trials demonstrate the potential challenges facing trial design in early-stage disease and emphasize the need to more aggressively pursue the discovery of reliable, user-friendly biomarkers to risk-stratify patients. This would be a significantly high-risk, high-reward effort that would require broad inter- and transdisciplinary collaboration, which would hopefully lead to more streamlined pragmatic adjuvant trials. A successful outcome in this space requires active participation among various disciplines, especially thoracic surgeons.

## References

[1] Shimkin MB Connelly RR Marcus SC Cutler SJ . Pneumonectomy and lobectomy in bronchogenic carcinoma. A comparison of end results of the Overholt and Ochsner clinics. J Thorac Cardiovasc Surg. (1962) 44:503–519. 13988545

[2] Jensik RJ Faber LP Milloy FJ Monson DO . Segmental resection for lung cancer: a fifteen-year experience. J Thorac Cardiovasc Surg. (1973) 66(4):563–572. 4356889

[3] Ginsberg RJ Rubinstein LV . Randomized trial of lobectomy versus limited resection for T1 N0 non-small cell lung cancer. Ann Thorac Surg. (1995) 60(3):615–623. doi: 10.1016/0003-4975(95)00537-U, PMID: 7677489

[4] Okada M Koike T Higashiyama M Yamato Y Kodama K Tsubota N . Radical sublobar resection for small-sized non–small cell lung cancer: a multicenter study. J Thorac Cardiovasc Surg. (2006) 132(4):769–775. doi: 10.1016/j.jtcvs.2006.02.063, PMID: 17000286

[5] Altorki N Wang X Kozono D Watt C Landrenau R Wigle D . Lobar or sublobar resection for peripheral stage IA non–small-cell lung cancer. N Engl J Med. (2023) 388:489–498. doi: 10.1056/NEJMoa2212083, PMID: 36780674 PMC10036605

[6] Saji H Okada M Tsuboi M Nakajima R Suzuki K Aokage K . Segmentectomy versus lobectomy in small-sized peripheral non–small-cell lung cancer (JCOG0802/WJOG4607L): a multicentre, open-label, phase 3, randomised, controlled, non-inferiority trial. Lancet. (2022) 399(10335):1607–1617. doi: 10.1016/S0140-6736(21)02333-3, PMID: 35461558

[7] Altorki NK Wang X Damman B Mentlick J Landreneau R Wigle D . Lobectomy, segmentectomy, or wedge resection for peripheral clinical T1aN0 non-small cell lung cancer: A post hoc analysis of CALGB 140503 (Alliance). J Thorac Cardiovasc Surg. (2024) 167(1):338–347. doi: 10.1016/j.jtcvs.2023.07.008, PMID: 37473998 PMC10794519

[8] Stinchcombe TE Wang X Damman B Mentlick J Landreneau R Wigle D . Secondary Analysis of the Rate of Second Primary Lung Cancer From Cancer and Leukemia Group B 140503 (Alliance) Trial of Lobar Versus Sublobar Resection for T1aN0 Non-Small-Cell Lung Cancer. J Clin Oncol. (2024) 42(10):1110–1113. doi: 10.1200/JCO.23.01306, PMID: 38215351 PMC11003504

[9] Darling GE Allen MS Decker PA Ballman K Malthaner RA Inculet RI . Randomized trial of mediastinal lymph node sampling versus complete lymphadenectomy during pulmonary resection in the patient with N0 or N1 (less than hilar) non–small cell carcinoma: results of the American College of Surgery Oncology Group Z0030 trial. J Thorac Cardiovasc Surg. (2011) 141(3):662–670. doi: 10.1016/j.jtcvs.2010.11.008, PMID: 21335122 PMC5082844

[10] Izbicki JR Passlick B Pantel K Pichlmeier U Hosch SB Karg O . Effectiveness of radical systematic mediastinal lymphadenectomy in patients with resectable non–small cell lung cancer: results of a prospective randomized trial. Ann Surg. (1998) 227(1):138–144. doi: 10.1097/00000658-199801000-00020, PMID: 9445122 PMC1191184

[11] Sugi K Nawata K Fujita N Ueda K Tanaka T Matsuoka T . Systematic lymph node dissection for clinically diagnosed peripheral non–small-cell lung cancer less than 2 cm in diameter. World J Surg. (1998) 22(3):290–295. doi: 10.1007/s002689900384, PMID: 9494422

[12] Altorki N Damman B Wang X Liberman M Wigle D Ashrafi A . The extent of lymph node dissection is not associated with disease-free survival following lobar or sublobar resection: Results from Cancer and Leukemia Group B 140503 (Alliance). J Thorac Cardiovasc Surg. (2025) 170(4):933–942. doi: 10.1016/j.jtcvs.2025.06.007, PMID: 40532782 PMC12289341

[13] Smeltzer MP Faris NR Ray MA Osarogiagbon RU . Association of pathologic nodal staging quality with survival in patients with non–small cell lung cancer after resection. JAMA Oncol. (2018) 4(1):80–87. doi: 10.1001/jamaoncol.2017.2993, PMID: 28973110 PMC5833630

[14] Altorki N Wang X Damman B Jones DR Wigle D Port J . Recurrence of Non-Small Cell Lung Cancer With Visceral Pleural Invasion: A Secondary Analysis of a Randomized Clinical Trial. JAMA Oncol. (2024) 10(9):1179–1186. doi: 10.1001/jamaoncol.2024.2491, PMID: 39088196 PMC11295064

[15] Stamatis G Leschber G Schwarz B Brintrup DL Flossdorf S Passlick B . Survival outcomes in a prospective randomized multicenter Phase III trial comparing patients undergoing anatomical segmentectomy versus standard lobectomy for non-small cell lung cancer up to 2 cm. Lung Cancer. (2022) 172:108–116. doi: 10.1016/j.lungcan.2022.08.013, PMID: 36058174

[16] Stamatis G Leschber G Schwarz B Brintrup DL Ose C Weinreich G . Perioperative course and quality of life in a prospective randomized multicenter phase III trial, comparing standard lobectomy versus anatomical segmentectomy in patients with non-small cell lung cancer up to 2 cm, stage IA (7th edition of TNM staging system). Lung Cancer. (2019) 138:19–26. doi: 10.1016/j.lungcan.2019.09.021, PMID: 31606521

[17] Jones DR Opitz I Harpole D Yanagawa J Lim E Tsutani Y . TROPION-Lung12: A phase 3 study of adjuvant datopotamab deruxtecan and rilvegostomig in ctDNA-positive or high-risk pathology stage I non-small cell lung cancer. J Thorac Cardiovasc Surg. (2026) 171(1):1–9. doi: 10.1016/j.jtcvs.2025.09.017, PMID: 40976544 PMC12536336

[18] Spigel DR Westeel V Anderson IC Greillier L Guisier F Bylicki O . Adjuvant chemotherapy for stage IA-IIA non-squamous, non-small-cell lung cancer identified as molecular high-risk by a 14-gene expression profile (AIM-HIGH): an international, randomised, phase 3 trial. Lancet Respir Med. (2025) 13(10):887–896. doi: 10.1016/S2213-2600(25)00213-9, PMID: 40578381

